# The matrix protein Fibulin-3 promotes KISS1R induced triple negative breast cancer cell invasion

**DOI:** 10.18632/oncotarget.25682

**Published:** 2018-07-10

**Authors:** Michelle M. Noonan, Magdalena Dragan, Michael M. Mehta, David A. Hess, Muriel Brackstone, Alan B. Tuck, Navin Viswakarma, Ajay Rana, Andy V. Babwah, Frederic E. Wondisford, Moshmi Bhattacharya

**Affiliations:** ^1^ Department of Physiology and Pharmacology, The University of Western Ontario, London, ON, Canada; ^2^ Department of Oncology, The University of Western Ontario, London, ON, Canada; ^3^ Department of Pathology, The University of Western Ontario, London, ON, Canada; ^4^ Lawson Health Research Institute, The University of Western Ontario, London, ON, Canada; ^5^ Division of Surgical Oncology, The University of Western Ontario, London, ON, Canada; ^6^ The Pamela Greenaway-Kohlmeier Translational Breast Cancer Research Unit, London Regional Cancer Program, London, ON, Canada; ^7^ Krembil Centre for Stem Cell Biology, Molecular Medicine Research Group, Robarts Research Institute, London, ON, Canada; ^8^ Department of Surgery, Division of Surgical Oncology, University of Illinois at Chicago, Chicago, IL, USA; ^9^ Department of Pediatrics, Child Health Institute of NJ, Rutgers-Robert Wood Johnson Medical School, New Brunswick, NJ, USA; ^10^ Department of Medicine, Child Health Institute of NJ, Rutgers-Robert Wood Johnson Medical School, New Brunswick, NJ, USA

**Keywords:** fibulin-3, kisspeptin receptor, triple-negative breast cancer, invasion, metastasis

## Abstract

Breast cancer is a leading cause of cancer mortality. In particular, triple negative breast cancer (TNBC) comprise a heterogeneous group of basal-like tumors lacking estrogen receptor (ERα), progesterone receptor (PR) and HER2 (ErbB2). TNBC represents 15–20% of all breast cancers and occurs frequently in women under 50 years of age. Unfortunately, these patients lack targeted therapy, are typically high grade and metastatic at time of diagnosis. The mechanisms regulating metastasis remain poorly understood. We have previously shown that the kisspeptin receptor, KISS1R stimulates invasiveness of TNBC cells. In this report, we demonstrate that KISS1R signals via the secreted extracellular matrix protein, fibulin-3, to regulate TNBC invasion. We found that the fibulin-3 gene is amplified in TNBC primary tumors and that plasma fibulin-3 levels are elevated in TNBC patients compared to healthy subjects. In this study, we show that KISS1R activation increases fibulin-3 expression and secretion. We show that fibulin-3 regulates TNBC metastasis in a mouse experimental metastasis xenograft model and signals downstream of KISS1R to stimulate TNBC invasion, by activating matrix metalloproteinase 9 (MMP-9) and the MAPK pathway. These results identify fibulin-3 as a new downstream mediator of KISS1R signaling and as a potential biomarker for TNBC progression and metastasis, thus revealing KISS1R and fibulin-3 as novel drug targets in TNBC.

## INTRODUCTION

Breast cancer is the most common cancer in women, worldwide [1]. Triple negative breast cancer (TNBC) is an aggressive breast cancer subtype, occurring often in women under 50 years of age or patients with BRCA1/BRCA2 mutation [2]. The lack of expression of estrogen and progesterone receptors (ER and PR) as well as the epidermal growth factor receptor 2 (HER2), renders TNBC resistant to hormonal and other targeted therapies [3]. Conventional chemotherapy is the standard of care with modest survival benefits in TNBC patients since patients initially respond well, but often develop chemoresistance [4]. TNBC patients have poor prognosis compared to other breast cancer subtypes. This can be attributed to the high incidence of disseminated tumor cells, leading to the onset of metastatic disease and associated morbidity [5–7]. To overcome this clinical problem, there is an urgent need to identify drug targets that are effective in treating metastatic TNBC.

Fibulin-3, also known as epidermal growth factor (EGF)-containing fibulin-like extracellular matrix protein 1 (EFEMP1), is a secreted glycoprotein found in the extracellular matrix (ECM) that mediates homotypic interactions between cells and heterotypic cell-matrix interactions and tissue remodeling [8]. Fibulin-3 is expressed in epithelial and endothelial cells where it is localized in the basement membrane [8, 9]. In the last decade, it has become clear that fibulin-3 can promote tumorigenesis. The expression of fibulin-3 is upregulated in metastatic stages of several carcinomas such as ovarian, cervical cancer, malignant gliomas and malignant mesothelioma and this upregulation has been linked to poor patient prognosis [10–13]. In fact, fibulin-3 has been proposed as a clinical biomarker for metastatic ovarian cancer [11] and malignant mesothelioma [14–16], since plasma levels are elevated in these cancer patients compared to healthy controls. Recent studies indicate that fibulin-3 may play a role in breast cancer. Fibulin-3 has been shown to regulate hypoxia-mediated breast cancer stem cell formation, a process which promotes primary tumor growth in animal xenograft models [17]. Moreover, fibulin-3 mRNA has been found to be overexpressed in pulmonary effusions from breast cancer patients [18]. Additionally, polymorphisms of the fibulin-3 gene might be associated with the risk of breast cancer in a Chinese sub-population [19]. However, whether fibulin-3 regulates TNBC metastasis is unknown.

The kisspeptin receptor (KISS1R) is a G-protein coupled receptor (GPCR) that binds kisspeptins (KPs), products of the *KISS1* gene. KPs (10, 13, 14 and 54 aa) are naturally-secreted, biologically-active, blood-borne peptides [20], derived from a pro-peptide that is cleaved rapidly by matrix metalloproteinases (MMPs) such as MT1-MMP, MMP-9 and furin to form KP-10 [21, 22]. All KPs have similar affinity for KISS1R [21], however, KP-10 is the agonist of choice for most studies [23–28]. KISS1R signals *via* a G_q/11_-coupled mechanism leading to the activation of phospholipase C and the subsequent activation of protein kinase C and ERK1/2 [29–31]. KISS1R has also been shown to activate ERK1/2 through a G-protein independent and β-arrestin2-dependent pathway [31, 32]. KISS1R signaling plays an important physiological role in the regulation of the reproductive axis and the initiation of puberty [33]. KISS1 and KISS1R (mRNA and protein) are expressed centrally and peripherally, including breast tissue [29, 34, 35]. *KISS1* (commonly classified as a metastasis suppressor gene) exerts anti-cancer roles in many cancers (reviewed [36]). However, when breast cells lose ERα, KISS1R signaling promotes epithelial-to-mesenchymal-transition (EMT) [37] and invasion by inducing invadopodia formation (*via* MT1-MMP [38]) and stimulating MMP-9 activity [39]. Recently, we have shown that KISS1R signaling promotes TNBC drug resistance [40]. In support of our findings, *mouse Kiss1r* has been shown to stimulate breast cancer metastasis in a mouse mammary tumor virus–polyoma virus middle T antigen model [41]. However, the mechanism by which KISS1R remodels the extracellular matrix for cell invasion is largely unknown. In this study, we demonstrate that the ECM protein fibulin-3 regulates TNBC metastasis in mouse models and signals downstream of KISS1R to stimulate TNBC cell migration and invasion, shedding light on whether TNBC cells employ KISS1R signaling via fibulin-3 to attain metastatic potential.

## RESULTS

### Plasma fibulin-3 levels in TNBC patients and healthy controls

**Figure 1:**
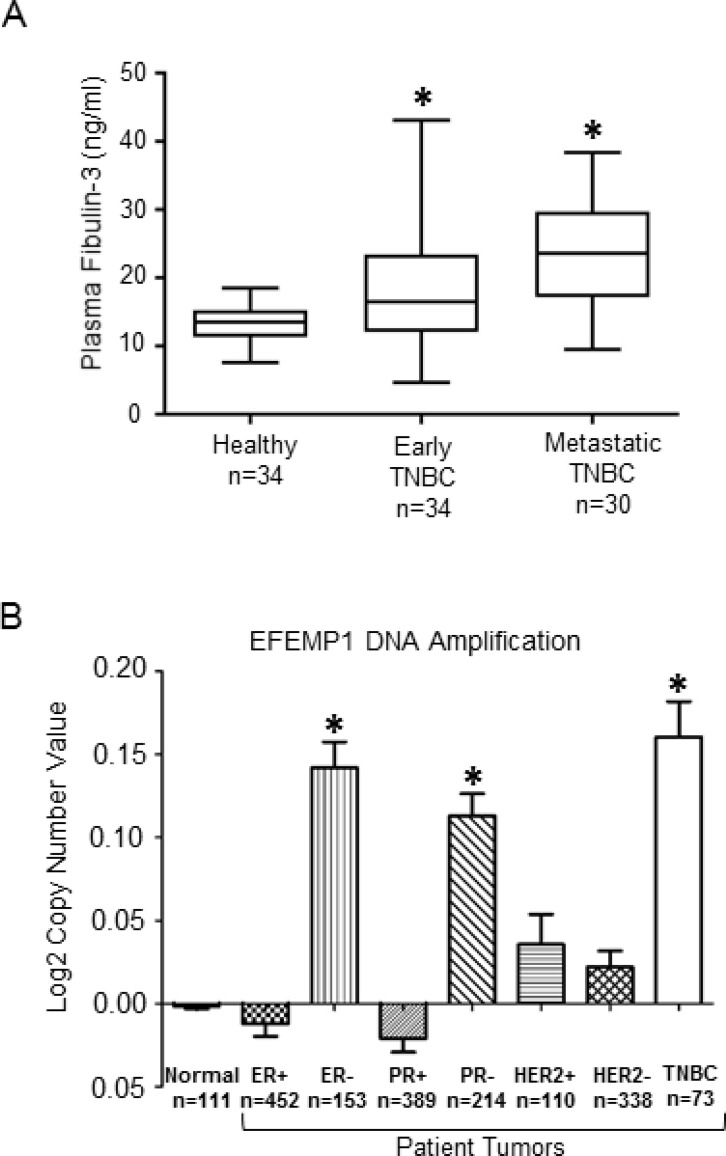
Fibulin-3 expression levels in TNBC patients (**A**) Plasma fibulin-3 levels (ng/ml) measured by ELISA in blood samples taken from healthy subjects (*n =* 34), non-metastatic TNBC patients (i.e. early disease; *n =* 34) or metastatic TNBC patients (*n =* 30). Statistical analysis done using Wilcoxon two-sample test with Bonferroni correction. Error bars: SD. (**B**) *EFEMP1* gene copy number observed in human breast cancer subtypes available through Oncomine dataset repository (www.oncomine.org). Data are log transformed and median centered (Y-axis).

Although fibulin-3 mRNA is overexpressed in effusions of human breast cancer patients [18], and fibulin-3 has been shown to promote breast tumor growth using animal models [17], whether plasma fibulin-3 levels differ in TNBC patients at different stage of disease is unknown. Thus, we measured plasma fibulin-3 concentrations by ELISA in TNBC patients (see Table 1 for patient demographics): newly diagnosed, non-metastatic TNBC (early disease), metastatic TNBC (advanced disease) and compared to healthy subjects (no prior history of breast cancer). We found that plasma fibulin-3 levels in TNBC patients were significantly higher (Figure 1A) compared to the levels observed in healthy females (metastatic: 23.5 ± 8.3 ng/ml; non-metastatic: 18.2 ± 7.7 ng/ml and healthy: 13.4 ± 3.1 ng/ml; *p =* 0.008 healthy vs. early; *p =* 0.010 early vs metastatic; *p <* 0.001 healthy vs metastatic). We also measured plasma fibulin-3 levels in non-TNBC patients, namely ER/PR-positive (HER2 negative) patients (Table 2, Supplementary Figure 1), and found that there was no significant difference in the plasma fibulin-3 levels in the non-TNBC patients (16.99 ± 5.8 ng/ml) compared to the levels observed in healthy females (14.45 ± 4.4 ng/ml). Interestingly, examination of breast cancer datasets using the Oncomine data repository (www.oncomine.org) revealed that the gene encoding fibulin-3, *EFEMP1* is amplified in TNBC patients (*n =* 73), in contrast to the expression in ERα-positive (*n =* 452) or HER2 positive (*n =* 110) patient tumors (Figure 1B).

**Table 1:** Clinical profile of study participants (females with TNBC) from London Health Science Centre

Characteristics	Number (range)	Fibulin-3 (ng/ml)
**Normal Subjects**		
Age (years)	Mean 32.8 (20–52)	13.4 ± 3.1
**Early disease TNBC patients** Age (years)	Mean 58.2 (27–89)	18.2 ± 7.7
Tumor size (mm)	Mean 27.1 (4–70)	
Blood collection		
before any treatment	20/34	
during/after chemotherapy	12/34	
after tumor removing surgery	7/34	
after radiation therapy	0/34	
Tumor size		
T1	16	
T2	10	
T3	3	
T4	5	
Node status		
N0	21	
N1	7	
N2	2	
N3	1	
Nx	3	
**Metastatic disease TNBC patients** Age (years)	Mean 60.9 (40–85)	23.5 ± 8.3
Surgery performed for tumor removal	24/30	
No surgery (primary tumor present)	6/30	
Chemotherapy at time of blood draw	25/30	
Radiation therapy at time of blood draw	23/30	
Site of metastasis		
brain	6	
lung	10	
bone	7	
lymph nodes	7	

**Table 2:** Clinical profile of study participants (non-TNBC females) from London Health Science Centre

Characteristics	Number (range)	Fibulin-3 (ng/ml)
**Normal Subjects**		
Age (years)	Mean 25.8 (22–55)	14.45 ± 4.4
**ER/PR Positive (HER2-negative) patients** Age (years)	Mean 69.4 (51–92)	16.99 ± 5.8
Tumor size (mm)	Mean 17.0 (6–45)	
Surgery performed for tumor removal	28/29	
No surgery (primary tumor present)	1/29	
hemotherapy	4/29	
Radiation therapy	19/29	
Tumor size		
T1	21	
T2	8	
T3	0	
T4	0	
Node status		
N0	22	
N1	2	
N2	0	
N3	0	
Nx	5	
Metastasis	0/29	

### Fibulin-3 knock-down decreases lung metastasis in a murine xenograft model

**Figure 2:**
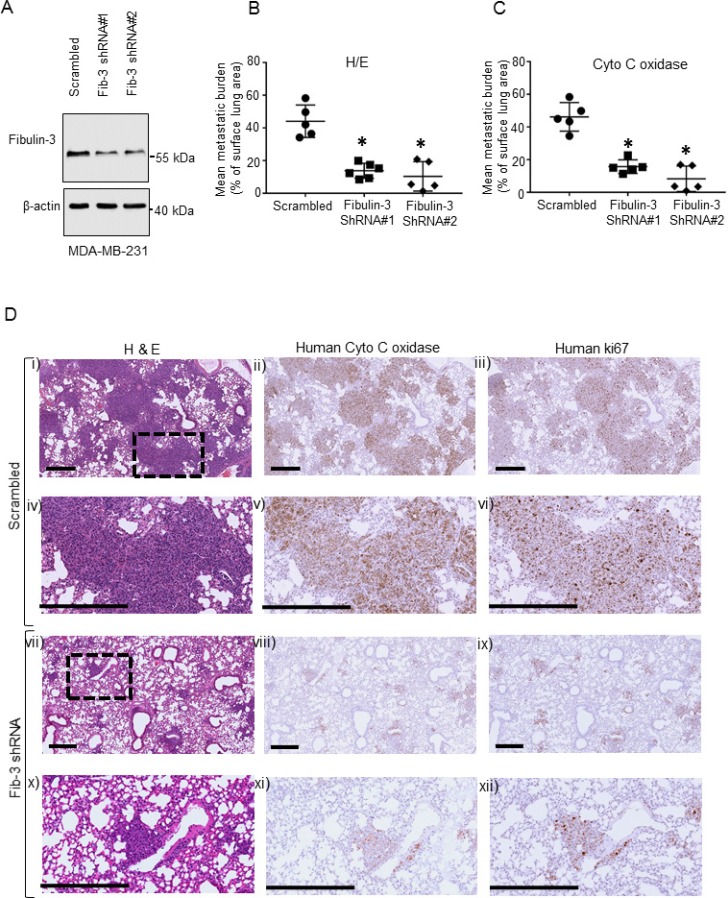
The effects of Fibulin-3 knockdown on TNBC metastasis (**A**) Representative western blot of fibulin-3 in human metastatic MDA-MB-231 cells stably expressing fibulin-3 shRNA or scrambled control. β-actin, loading control. Densitometric analysis of blots shown in Supplementary Figure 2A (*n =* 3). (**B**, **C**) Fibulin-3 downregulation reduces lung colonization in a xenograft experimental metastasis mouse model. Lung metastases formed by triple negative MDA-MB-231 cells expressing scrambled control or fibulin-3 shRNA (*n =* 5–6 mice per group). Points represent lung metastatic burden from each mouse and bars represent mean surface area ± SEM. Mean metastatic burden was quantified blindly in (B) hematoxylin and eosin and (C) human mitochondrial enzyme (anti-cytochrome c oxidase) stained slides. (**D**) Representative images from xenografts showing lung tissue sections subjected to either hematoxylin and eosin (left column), or human anti-cytochrome c oxidase staining (middle column) or anti-human Ki67 staining (right column). Boxed area (inset) in each upper panel (i, ii, iii) and (vii, viii, ix) is magnified in the corresponding lower panel (iv, v, vi) and (x, xi, xii), respectively. Scale bar, 400 μm.

Since our clinical data revealed that plasma fibulin-3 concentrations were significantly elevated in patients with TNBC metastatic tumors, we investigated whether human fibulin-3 expression affects metastasis using a xenograft model of experimental metastasis in NOD/SCID/IL2 receptor γ null mice [42]. Endogenous fibulin-3 was significantly reduced in the metastatic TNBC MDA-MB-231 cells using lentivirus-delivered shRNA and knockdown was verified by western blot and qPCR (Figure 2A, Supplementary Figure 2A, 2C). The expression of fibulin-3 shRNA did not affect cell viability (Supplementary Figure 2E). Following tail vein injection, metastatic tumor burden in the lung (percentage of lung occupied by tumor) was significantly lower in mice injected with human metastatic MDA-MB-231 cells expressing fibulin-3 shRNA, compared to mice injected with scrambled controls (Figure 2B–2D). Boxed area (inset) in each upper panel (i, ii, iii) and (vii, viii, ix) is magnified in lower panels (iv, v, vi) and (x, xi, xii), respectively. Human tumor cells colonized in the lungs were identified using a human mitochondrial antibody [43] (Figure 2D, middle column panel) and also using an anti-human Ki67, a cell proliferation marker (Figure 2D, right column panel). This suggests that there is a reduction in the number of human tumor cells present in the fibulin-3 knockdowns. Taken together, these results reveal that fibulin-3 expression regulates human TNBC tumor growth and colonization *in vivo*.

### Fibulin-3 downregulation reduces human TNBC cell migration and invasion

**Figure 3:**
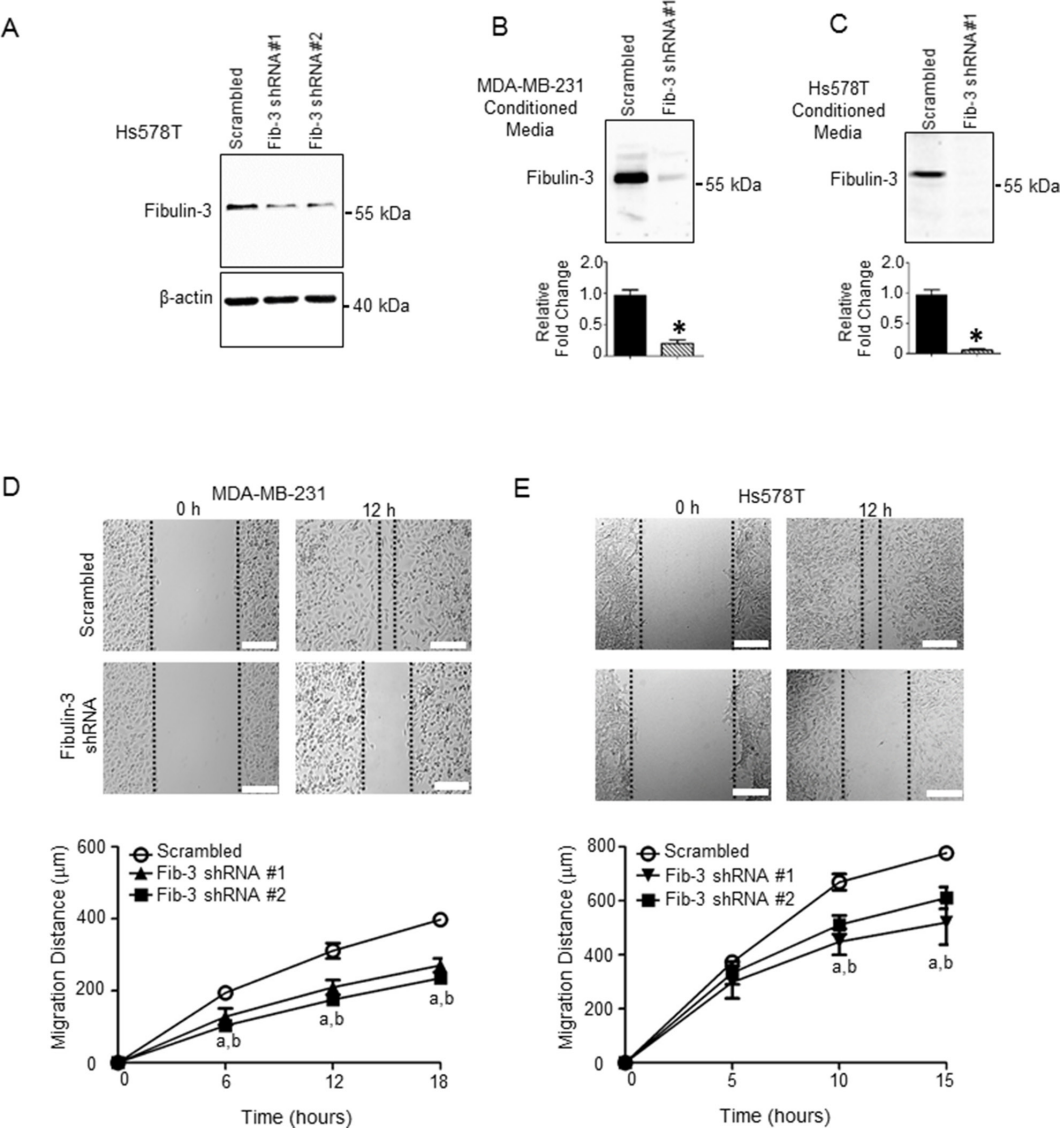
Fibulin-3 knockdown in TNBC cells decreases TNBC cell motility (**A**) Representative western blot showing the expression of fibulin-3 in Hs578T cells stably expressing fibulin-3 shRNA or scrambled control; β-actin, loading control. See Supplementary Figure 2B for densitometric analysis of blots (*n =* 3). Representative western blot showing fibulin-3 expression in conditioned media from (**B**) MDA-MB-231 and **(C)** Hs578T cells stably expressing fibulin-3 shRNA or scrambled control. Densitometric analysis of blots shown below. Student’s *t*-test: ^*^, *P <* 0.05. Bars represent protein expression ± SEM (*n =* 5). Cell motility measured using the scratch assay in (**D**) MDA-MB-231 and (**E**) Hs578T cells expressing fibulin-3 shRNA vs. scrambled control (*n =* 4). Two-way ANOVA followed by Bonferroni’s multiple comparison test: a, *P <* 0.05 for fibulin-3 shRNA construct #1 vs. scrambled control; b, *P <* 0.05 for fibulin-3 shRNA construct #2 vs. scrambled control. Scale bar = 50 μm.

The ability of cancer cells to metastasize is dependent on the cell’s capacity to degrade and invade the surrounding tissue, as well as its ability to migrate away from the primary site of tumor formation. To examine a potential mechanism by which fibulin-3 regulates TNBC metastasis, we determined the effect of fibulin-3 knock-down on cell migration and invasion in two TNBC cell lines, MDA-MB-231 and Hs578T. In both cell lines, stable expression of fibulin-3 shRNA constructs significantly diminished fibulin-3 mRNA and protein expression (Figures 2A, 3A, Supplementary Figure 2A–2D) as well as fibulin-3 secretion (Figure 3B, 3C). Expression of shRNA constructs had no effect on cell viability (Supplementary Figure 2E, 2F). TNBC cells expressing fibulin-3 shRNA demonstrated a significant decrease in migration compared to scrambled controls in a scratch assay (Figure 3D, 3E; Supplementary Movie 1). To determine whether expression of fibulin-2 shRNA constructs altered cell proliferation, we conducted cell growth assays (Supplementary Figure 2G, 2H) and found no differences in cell proliferation. Thus, the observed differences in migration by scratch wound closure and transwell migration assays were a result of changes in cell migration and not due to differences in cell proliferation.

**Figure 4:**
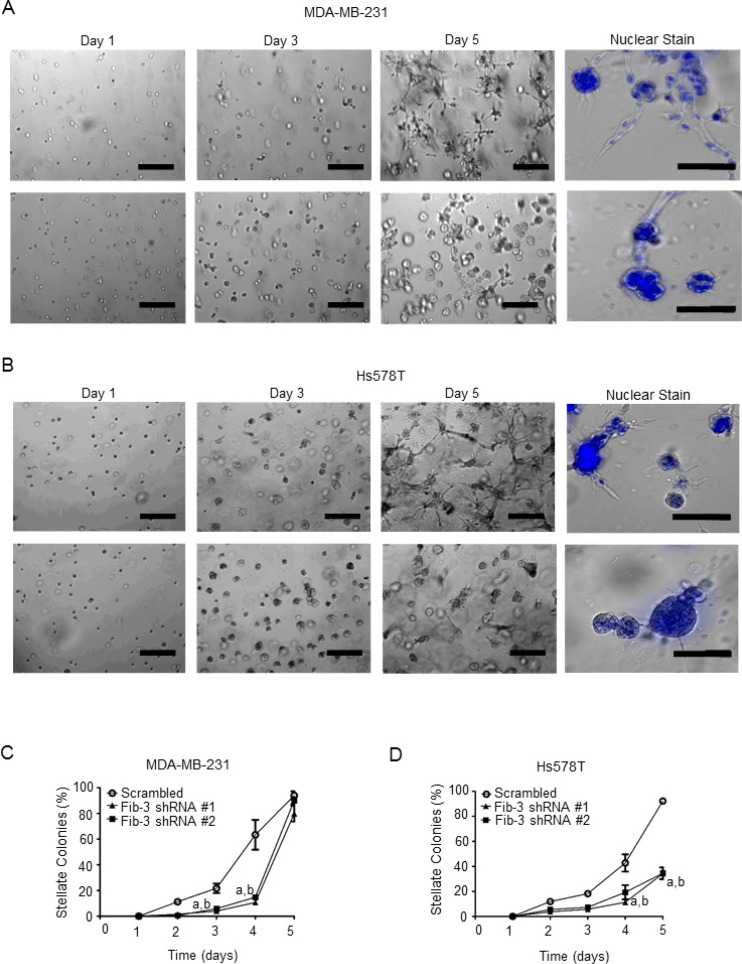
Fibulin-3 knockdown inhibits TNBC cell invasion in three-dimensional culture models (**A**) MDA-MB-231 cells or (**B**) Hs587T cells expressing fibulin-3 shRNA or scrambled controls were suspended in Matrigel for 5 days and the number of invasive stellate structures were counted (*n =* 4). Cells were fixed and nuclei were stained with Hoechst 33242 on day 5. Representative images shown. Scale bar = 50 μm. Quantification of invasive stellate structures in each cell line is shown in (**C**) and (**D**). Two-way ANOVA followed by Bonferroni’s multiple comparison test: a, *P <* 0.05 scrambled control vs fibulin-3 shRNA construct #1; b, *P <* 0.05 scrambled control vs fibulin-3 shRNA construct #2.

Matrigel is a reconstituted extracellular matrix that mimics the *in vivo* tumor microenvironment [44, 45]. To assess a role for fibulin-3 in TNBC cell invasion, a three-dimensional Matrigel invasion assay was conducted, as previously described [37, 39, 44, 46]. MDA-MB-231 and Hs578T cells expressing fibulin-3 shRNA had a diminished ability to form invasive (stellate) colonies compared to scrambled control cells (Figure 4A–4D). The viability of colonies was verified using Hoechst staining (shown in blue) to examine cell nuclear integrity (Figure 4A, 4B, right panels).

### Fibulin-3 mediates KISS1R-induced TNBC cell migration and invasion

**Figure 5:**
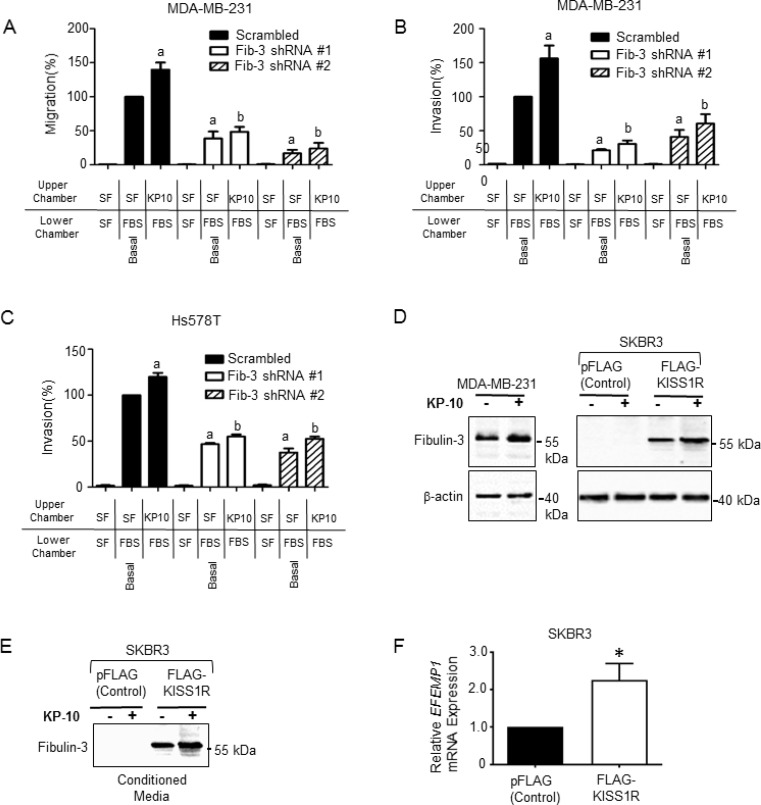
Fibulin-3 knock-down inhibits kisspeptin induced TNBC cell migration and invasion (**A**) MDA-MB-231 cells expressing fibulin-3 shRNA display decreased migration towards 10% fetal bovine serum (FBS) under basal and KP-10 stimulated conditions (*n =* 3). One-way ANOVA followed by Bonferroni’s multiple comparison test: a, *P <* 0.05 for significance difference vs scrambled control non-stimulated; b, *P <* 0.05 for significance difference vs scrambled control 100 nM kisspeptin-10. Bars represent cells migrated to lower chamber ± SEM, as percentage of control. (**B**) MDA-MB-231 cells and (**C**) Hs578T cells depleted of fibulin-3 display decreased invasion towards 10% FBS under basal conditions and KP-10 induced conditions (*n =* 3). One-way ANOVA followed by Bonferroni’s multiple comparison test: a, *P <* 0.05 for significance difference vs scrambled control non-stimulated; b, *P <* 0.05 for significance difference vs scrambled control 100 nM kisspeptin-10. Bars represent cells invaded to lower chamber ± SEM, as percentage of control. (**D**) KP-10 stimulates the expression of fibulin-3 in ERα-negative breast cancer cells. Representative western blot showing expression levels of fibulin-3 in cells stimulated with KP-10 (100 nM, 72 hours). (**E**) KP-10 stimulates the secretion of fibulin-3 in ERα-negative SKBR3 breast cancer cells. Representative western blot of cell lysates showing fibulin-3 in conditioned media from ER-α negative SKBR3 breast cells (100 nM, 24 hours). (**F**) Relative mRNA expression of fibulin-3 gene, *EFEMP1* by RT-qPCR in SKBR3 cells stably expressing KISS1R and pFLAG vector controls. Columns represent mean relative mRNA expression, normalized to GAPDH ± SEM; student’s unpaired *T*-test: ^*^P < 0.05. (*n* = 5). See Supplementary Figure 3A–3C for densitometric analysis of blots.

KISS1R has been implicated in promoting breast cancer metastasis *in vivo* [41], and kisspeptin-10 (KP-10) mediated activation of KISS1R has been shown to stimulate TNBC MDA-MB-231 and Hs578T cell invasion [39]; these cells express endogenous KISS1R [38]. However, the molecular mechanisms by which KP/KISS1R signaling stimulates TNBC cell invasion are largely unknown. Having shown that fibulin-3 expression regulates invasiveness, we next investigated whether fibulin-3 mediates KISS1R-induced TNBC cell migration and invasion using transwell chamber assays, as previously described [37, 39, 44, 46]. MDA-MB-231 and Hs578T cells expressing fibulin-3 shRNA or scrambled control were seeded in serum-free media, or serum-free media containing 100 nM KP-10 (based on dose response studies [39]). Downregulation of fibulin-3 inhibited the basal and KP-10-induced TNBC cell migration (Figure 5A) and invasion (Figure 5B, 5C). These findings indicate that fibulin-3 regulates TNBC invasiveness, downstream of KISS1R activation.

### KISS1R signaling stimulates fibulin-3 expression and secretion in ERα-negative breast cancer cells

Next, to investigate if KISS1R activation promotes fibulin-3 expression, ERα-negative breast cancer cells were treated with 100 nM KP-10 and fibulin-3 protein expression in cell lysates, and in conditioned media was examined by western blot analysis. Treatment of MDA-MB-231 cells with KP-10 (100 nM, 72 hr) increased fibulin-3 protein levels compared to unstimulated cells (Figure 5D, Supplementary Figure 3A). We have previously shown that ERα-negative SKBR3 breast cancer cells express low levels of endogenous KISS1R [37, 40], and stable over-expression of FLAG-KISS1R promoted an EMT-like event, resulting in increased tumor cell invasion [37]. Here, we observed that KP-10 treatment also increased fibulin-3 protein levels in FLAG-KISS1R SKBR3 cells (Figure 5D, Supplementary Figure 3B) and stimulated fibulin-3 secretion (Figure 5E, Supplementary Figure 3C), compared to untreated controls. The increase in fibulin-3 protein levels in FLAG-KISS1R SKBR3 cells is likely due to an increase in *EFEMP1* mRNA expression in these cells, compared to controls (Figure 5F). These results show that KISS1R signaling regulates fibulin-3 expression and secretion in ERα-negative breast cancer cells.

### Downregulation of fibulin-3 decreases MMP-9 expression and activity

**Figure 6:**
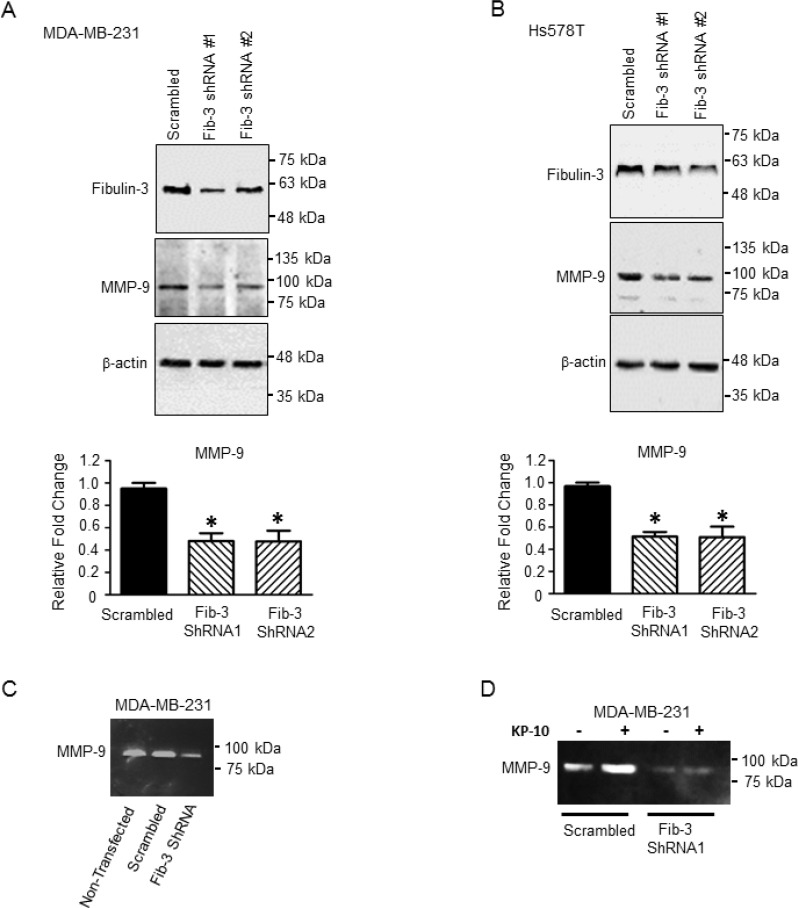
Downregulation of fibulin-3 in TNBC cells decreases the expression and activity of MMP-9 Representative western blot and densitometric analysis of MMP-9 in (**A**) MDA-MB-231 and (**B**) Hs578T cells expressing fibulin-3 shRNA or scrambled control; β-actin, loading control (*n =* 3). One-way ANOVA followed by Dunnett’s multiple comparison test: ^*^, *P <* 0.05 compared to scrambled. Bars represent protein expression ± SEM. (**C**) Downregulation of fibulin-3 in MDA-MB-231 cells reduces MMP-9 activity (*n =* 4) shown by zymography. (**D**) Downregulation of fibulin-3 in MDA-MB-231 cells blocks basal and KP-10 stimulated MMP-9 secretion and activity (*n =* 4).

MMP-9 is over-expressed in TNBC tumors and this is associated with a higher incidence of metastasis in patients [47]. Serum levels of MMP-9 were found to be high in breast cancer patients compared to healthy subjects [48]. Fibulin-3 has been shown to regulate MMP-9 expression and activity in lung cancer and malignant glioma [13, 49]. Therefore to determine whether fibulin-3 regulates TNBC cell invasion *via* MMP-9, we examined the effect of fibulin-3 downregulation on MMP-9 expression and activity by western blotting and zymography, respectively. MMP-9 expression significantly decreased upon downregulation of fibulin-3 in both MDA-MB-231 and Hs578T cell lines compared to scrambled controls (Figure 6A, 6B). Additionally, zymography demonstrated that MMP-9 activity was diminished in the MDA-MB-231 cells expressing fibulin-3 shRNA (Figure 6C). We have previously shown that KP-10 stimulates MMP-9 secretion and activity in MDA-MB-231 cells [39]. Thus, we sought to determine if fibulin-3 regulates KP-10 induced MMP-9 activity. Our results demonstrate that MDA-MB-231 cells expressing fibulin-3 shRNA had diminished basal and KP-10 induced secretion and activity of MMP-9 (Figure 6D). This suggests that KISS1R signaling stimulates MMP-9 secretion and activity *via* a fibulin-3 dependent mechanism.

### Fibulin-3 regulates KP-10 induced MMP-9 activity *via* MAPK signaling

**Figure 7:**
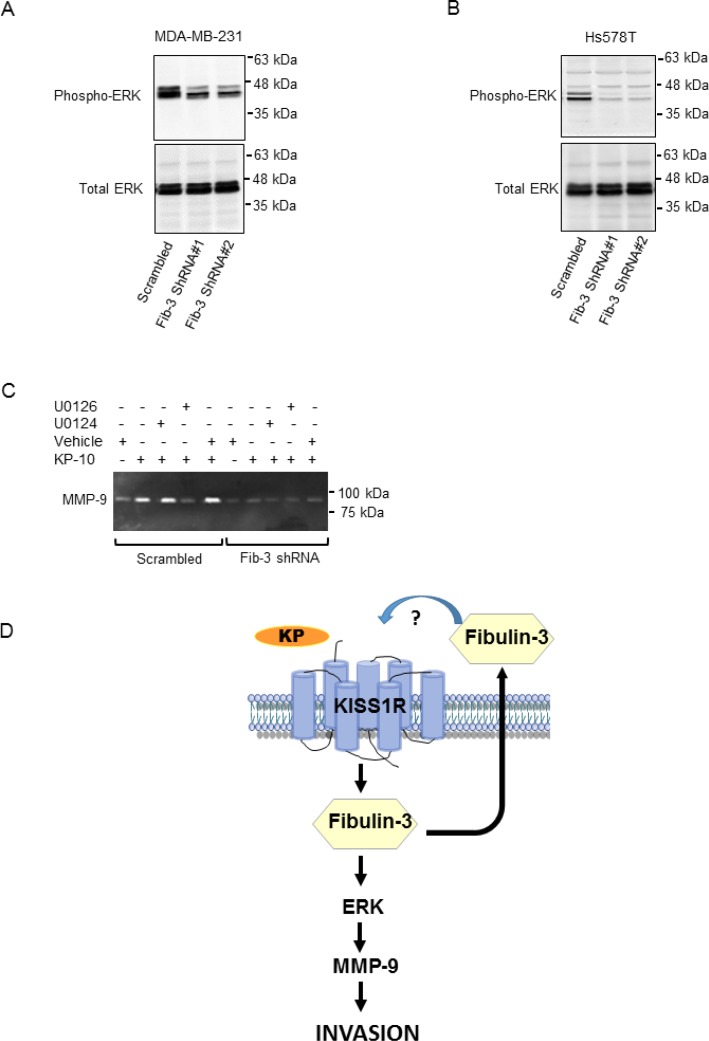
Downregulation of fibulin-3 in TNBC cells reduces ERK1/2 phosphorylation Representative western blot of pERK and total ERK in (**A**) MDA-MB-231 cells or (**B**) Hs578T cells expressing fibulin-3 shRNA or scrambled control; total ERK, loading control (*n =* 3). (**C**) Inhibition of ERK signaling blocks basal and KP-10-induced MMP-9 secretion and activity. MDA-MB-231 cells stably expressing fibulin-3 shRNA or scrambled control were pre-treated for 30 minutes with the following drugs prior to stimulation with 100 nM KP-10 for 24 hours: 20 μM active ERK1/2 inhibitor, U0126 or 20 μM inactive analog, U0124. Representative zymograph of three independent experiments is shown. (**D**) Proposed model of KISS1R induced invasion in TNBC: KISS1R signaling stimulates fibulin-3 expression and secretion. Fibulin-3 promotes TNBC cell invasion through ERK activation and MMP-9 secretion and activity.

We have shown that KISS1R signaling stimulates the phosphorylation of the mitogen-activated protein kinases (MAPK), ERK1/2, to regulate TNBC invasion [38]. Fibulin-3 regulates the phosphorylation of ERK and AKT in pancreatic cancer cells [50]. To examine the potential mechanisms by which fibulin-3 regulates TNBC cell invasion, we investigated the phosphorylation status of ERK and AKT in the presence of high vs. low levels of fibulin-3. Upon downregulation of fibulin-3 in the TNBC cell lines, we observed decreased phosphorylation of ERK compared to scrambled controls (Figure 7A, 7B, Supplementary Figure 3D, 3E). However, there was no significant change in AKT phosphorylation in these cells (Supplementary Figure 4A, 4B).

Next, we investigated whether or not inhibiting ERK1/2 phosphorylation impaired KP-10 induced MMP-9 activity via MAPK, downstream of fibulin-3. We observed an inhibition of KP-10-dependent MMP-9 secretion and activity in cells treated with the ERK1/2 inhibitor, U0126 compared to cells treated with the inactive analog U0124 (Figure 7C). However, in fibulin-3 depleted cells, KP-10 failed to stimulate MMP-9 activity in the presence or absence of the ERK inhibitor, indicating that fibulin-3 and ERK1/2 act as downstream mediators of KP/KISS1R signaling in TNBC cell invasion (Figure 7D). Overall, this data suggests that fibulin-3 regulates KP-10 induced TNBC cell invasion *via* an ERK and MMP-9 dependent pathway.

## DISCUSSION

Metastasis attributes to more than 90% of breast cancer-related deaths [51]. The tumor microenvironment has been shown to drive metastasis [52]. Therefore, components of the tumor microenvironment including ECM proteins are emerging as therapeutic targets in the treatment of metastasis [51, 53, 54]. Specifically, proteins secreted by cancer cells into the tumor microenvironment may represent important targets, as their expression profiles have the potential to aid in earlier diagnosis and the assessment of cancer progression [55].

Fibulin-3 is a component of the ECM and during development, fibulin-3 binds to the elastin precursor, tropoelastin and regulates elastic fiber assembly [9]. Mutations in the fibulin-3 gene have been associated with hernias and macular degenerative diseases, most likely due to reduced elastic fiber formation in these organs [56, 57]. Structurally, fibulins have tandemly repeated EGF-like domains, as well as a distinctive C-terminal fibulin domain [57]. In the last decade, it has become clear that fibulin-3 appears to play a dual role in cancer depending on different cancer-cell contexts, promoting tumorigenesis in some cancers [10–13] or having anti-tumor effects in others [58]. For example, the tumor suppressive role of fibulin-3 has been attributed to downregulated expression of the fibulin-3 gene due to hypermethylation in lung, colorectal and liver cancers [59–61]. Fibulin-3 has also been shown to suppress growth signaling in nasopharyngeal carcinomas and glioma [62, 63], and can inhibit cell migration and MMP-induced cancer cell invasion in other cancers [58, 62, 64].

The oncogenic roles of fibulin-3 have been shown to be related to the increased expression of MMP-2 via NF-kappaB activation in osteosarcoma [65], activation of AKT and MAPK pathways in pancreatic cancer [50], activation of NOTCH signaling in gliomas [66] and regulation of insulin-like growth factor-binding protein-5 expression in bladder cancer metastasis [67]. A recent study revealed that fibulin-3 plays a role in breast cancer, and showed that downregulation of fibulin-3 in human TNBC Hs578T cells decreased primary tumor growth in an orthotopic xenograft model, by impairing the tumor initiation potential of breast cancer stem cells, downstream of hypoxia inducible factor (HIF2α) [17]. Here we demonstrate for the first time that fibulin-3 promotes TNBC metastasis, since the downregulation of fibulin-3 in TNBC cells impaired lung colonization in an experimental metastasis model. As is the case with several other tumor-promoting genes, we found in examining human breast cancer datasets using Oncomine that the fibulin-3 gene *EFEMP1* is often amplified in TNBC patients. Furthermore, our results suggest that fibulin-3 detection in the plasma maybe used to discriminate between healthy individuals and patients with TNBC, despite the small sample size in each cohort and thus could potentially serve as an adjunct in diagnosis. Although the significance of *EFEMP1* gene amplification in TNBC is currently unknown, it may be a possible mechanism by which fibulin-3 is overexpressed [18]. Interestingly, *EGFR* gene is amplified in TNBC [68–70], and high *EGFR* copy number was found to be associated with poor clinical outcome of the patients [70]. Further studies are required to determine whether *EFEMP1* copy number is altered in TNBC and whether this is beneficial for predicting patient outcomes.

ECM remodeling, migration and invasion are all integral to the process of cancer cell metastasis. Downregulation of fibulin-3 resulted in decreased basal and KP-10 stimulated TNBC cell migration and invasion. We have previously shown that KISS1R signaling stimulates TNBC invasion via MMP-9 [39]. MMP-9 promotes cell invasion downstream of ERK in breast cancer cells [71]. We found that a reduction in fibulin-3 levels resulted in decreased phosphorylation of ERK, and reduced expression and activity of MMP-9. Thus, our results suggest that fibulin-3 promotes basal and KISS1R-induced TNBC cell invasion by regulating MMP-9 secretion and activity via ERK (Figure 7D).

We also found that KISS1R signaling regulates the mRNA and protein expression and secretion of fibulin-3, supporting that fibulin-3 may be a downstream effector of KISS1R signaling; this suggests that KISS1R signaling may influence components of the tumor microenvironment via fibulin-3. In fact, KISS1 and KISS1R have been shown to be highly expressed in mesenchymal stem cells in multiple myeloma [72]. Whether KISS1, KISS1R and fibulin-3 are expressed in the TNBC tumor microenvironment has yet to be determined. Additionally, further studies are required to delineate an intracellular *versus* extracellular role for fibulin-3 in mediating KISS1R signaling, as well as the mechanisms regulating the kisspeptin-dependent expression and secretion of fibulin-3. One possible mechanism by which KISS1R regulates *EFEMP1* expression could be *via* MARK [73]. KISS1R associates with the actin protein IQGAP1 [37] shown to regulate protein secretion *via* the exocyst complex [74]. Whether KISS1R signaling regulates fibulin-3 secretion via IQGAP1 remains to be tested. Fibulin-3 associates with several endoplasmic reticulum proteins in retinal cells that can regulate its secretion [75], however, a role for KISS1R in this pathway remains to be elucidated. In conclusion, our findings support the notion that fibulin-3 is a novel and promising new biomarker for TNBC. Given the scarce treatment options available to TNBC patients, this study highlights fibulin-3 and KISS1R as potential targets for the prevention of TNBC metastasis.

## MATERIALS AND METHODS

### Blood collection and Fibulin-3 ELISA

The study was approved by the Office of Human Research Ethics, Western University, and all female participants provided informed consent. Blood (5 mL) was collected in BD Vacutainer K2 EDTA tubes (VWR International; Radnor, PA, USA) from the following groups: healthy subjects group 1 (*n =* 34), non-metastatic TNBC patients (*n =* 34), metastatic TNBC patients (*n =* 30) healthy subjects group 2 (*n =* 27), ER/PR-positive (HER-2 negative) breast cancer patients (i.e non-TNBC, *n =* 29) presenting to the Breast Care Clinic at St. Joseph’s Health Care London or at the London Regional Cancer Program. Blood was centrifuged at 3000 rpm for 10 min, and the plasma was collected and frozen immediately in liquid nitrogen. Samples were stored at −196° C and subsequently thawed to quantify plasma fibulin-3 concentrations using an enzyme-linked immunosorbent assay (ELISA) kit (USCN Life Science Inc.; Wuhan, Hubei, China) according to manufacturer’s instructions [15]. Absorbance was measured at 450 nm using a Victor 3V Multi-Detection Microplate Reader (PerkinElmer; Waltham, MA, USA). Statistical analysis of gene expression in clinical samples was conducted by a biostatistician (Statistical Services, Western) using a Wilcoxon two-sample test with Bonferroni correction.

### Cell culture

Human breast cell lines were purchased from American Type Culture Collection (Manassas, VA, USA) and were maintained at 37° C with 5% CO_2_. MDA-MB-231, Hs578T and SKBR3 were cultured in RPMI 1640 supplemented with 10% (v/v) fetal bovine serum (FBS).

Gene knockdown of fibulin-3 was achieved using lentivirus-delivered pGFP-C-shLenti shRNA cloning plasmid (Origene Technologies; Rockville, MD, USA). Heterogeneous populations of stable transfectants were selected using medium containing puromycin (1.5 µg/mL). Similarly, cells expressing scrambled controls were also generated and fibulin-3 expression in cells was verified weekly by Western blot analysis. SKBR3 cell lines stably expressing KISS1R (SKBR3 FLAG-KISS1R) and pFLAG vector controls were generated as described [37] and represent polyclonal cell populations; these were grown in media containing G418 (1 µg/mL) and KISS1R over-expression was verified weekly by Western blot analysis.

### Xenograft mouse model: experimental metastasis assay

Xenograft experiments were conducted as previously described [76]. The human metastatic triple negative MDA-MB-231 cells expressing scrambled control or two individual fibulin-3 shRNA constructs were re-suspended in PBS (5 × 10^5^ cells/mouse) and injected into the tail vein of 6-week-old female NOD/SCID-IL2Rγ null (immunocompromised) mice. At 3 weeks post-injection, mice were sacrificed and lung tissues were harvested, fixed in 4% paraformaldehyde and embedded in paraffin. Sections were stained as previously described [42] using hematoxylin and eosin, anti-human Cytochrome c oxidase subunit II (1:100 Abcam) and anti-human Ki67 (1:100 dilution, Fischer Scientific). Lung metastatic tumor burden was quantified blindly in lung sections stained with hematoxylin/eosin, and anti-human Cytochrome c oxidase using Aperio ImageScope software. Slides were reviewed by pathologist (Dr A. Tuck, London Health Sciences Center). All animal procedures were conducted in accordance with the recommendations of the Canadian Council on Animal Care, under a protocol approved by the Western University Animal Care Committee.

### Immunoblot assays

Experiments were performed previously as described [37, 39]. Cells were lysed using RIPA buffer and protein was separated by SDS-PAGE. Protein (50 µg) levels were quantified using antibodies raised against human proteins: rabbit anti-fibulin-3 (1:1000; Abcam), rabbit anti-KISS1R (1:4000, Abcam), rabbit anti-MMP-9 (1:500; Abcam), rabbit anti-ERK1/2 (1:1000; Cell Signaling), rabbit anti-phoshpo-ERK1/2 (1:2000; Cell Signaling), rabbit anti-AKT (1:1000; Cell Signaling), rabbit anti-phospho-AKT (1:1000; Cell Signaling). β-Actin or glyceraldehyde 3-phosphate dehydrogenase (GAPDH) expression was used as a loading control and was examined using rabbit anti-Actin (1:5000; GeneTex Inc.) or mouse anti-GAPDH (1:3000; GeneTex Inc.). After 1-hour incubation with horseradish peroxidase (HRP)-conjugated secondary antibodies, rabbit (1:2500, GE Healthcare) or mouse (1:2500, GE Healthcare), the proteins were visualized using SuperSignal West Dura Extended Duration Substrate (Thermo Scientific) and a VersaDoc Imaging System (Bio-Rad).

To determine the effect of KP-10 treatment on fibulin-3 expression, cells were treated with KP-10 (100 nM) (Calbiochem) in FBS-supplemented media for 72 hours, where applicable.

### Immunoblot analysis of Fibulin-3 secretion

Triple negative breast cancer cells (TNBC) MDA-MB-231 and Hs578T cells (3 × 10^6^) expressing fibulin-3 shRNA or scrambled controls were plated in a 10 cm culture dish, serum starved for 24 hours and then conditioned media were collected. SKBR3 pFLAG and FLAG-KISS1R cells (3 × 10^6^) were also plated in 10 cm culture dishes, serum starved for 24 hours, stimulated with 100 nM KP-10 for 24 hours and then conditioned media were collected. Conditioned media were concentrated using Microsep™ 10 K Advance Centrifugal Filters (Pall Life Sciences) and centrifuged at 5000 rcf at 4° C for 30 minutes. Protein (75 µl) was separated by SDS-PAGE and secreted fibulin-3 levels were quantified with rabbit anti-fibulin-3. Ponceau stain was used to assess the equal loading amount of total protein.

### Scratch assays

These assays were conducted as previously described [37, 38, 40]. MDA-MB-231 and Hs578T cells expressing fibulin-3 shRNA or scrambled control, plated in duplicate wells and grown to confluence in a 12-well plate, were scratched with a sterile pipette tip. Cells in FBS supplemented media were allowed to migrate into the scratch for 18 hours and were imaged every 15 minutes using an automated Olympus IX-81 microscope (Olympus; Shinjuku, Tokyo, Japan). Distance travelled was measured and analyzed using *In Vivo* Analyzer Suite (Media Cybernetics; Rockville, MD, USA) and this software was used for time-lapse microscopy (see Supplementary Movie). The width of the scratch (μm) was measured at seven points along the scratch for each image taken (per time point). The distance migrated was calculated by subtracting the width of the scratch at each time point from the width of the scratch at time zero. The distances migrated into the scratch at each of the seven points/image was averaged to determine the distance migrated for each well, as described [40].

### Transwell chamber cell migration and invasion assays

Transwell chamber migration and Matrigel invasion assays were conducted as previously described [37–39], with MDA-MB-231 and Hs578T cells expressing fibulin-3 shRNA or scrambled control. For invasion assays, cells were layered on top of a 1:10 dilution of Matrigel (8.0 mg/mL; BD Biosciences) dissolved in serum-free media. Cells were then fixed with a 20% acetone: 80% methanol solution and nuclei stained with 0.1% Hoechst 33258 (Invitrogen). Two replicates were conducted for each condition and 24 fields per condition were imaged using an Olympus IX-71 inverted microscope (Olympus). Cells were counted using *In Vivo* Analyzer Suite (Media Cybernetics; Rockville, MD, USA).

### Three-dimensional (3D) invasion assays

3D invasion assays were conducted as described previously [37, 39]. MDA-MB-231 and Hs578T cells (2.5 × 10^4^) expressing fibulin-3 shRNA or scrambled control were seeded in a 1:1 dilution of phenol red-free Matrigel (8.0 mg/mL; BD Biosciences) and culture medium on Matrigel-coated 35 mm glass-bottom culture dishes (MatTek). Cultures were maintained and imaged daily for 5 days with an Olympus IX-81 microscope, using *In Vivo* Analyzer Suite. Cell colonies were scored blindly as being either stellate or spheroidal. A colony was deemed to be stellate if one or more projections from the central sphere of a colony of cells was observed.

### PCR

Reverse-transcription was carried out according to manufacturer’s instructions using iScript RT Supermix (Bio-Rad). Gene expression was determined using SYBR green real-time qPCR (RT-qPCR) as previously described [40]. The steady-state mRNA levels of each gene of interest were determined by amplification of cDNA using specific primers against the fibulin-3 gene, as described [77] and results were normalized to *GAPDH*.

### Zymography

Zymographic analysis was performed as described previously [39, 78]. MDA-MB-231 cells expressing fibulin-3 shRNA or scrambled control and SKBR3 cells expressing pFLAG and FLAG-KISS1R vectors (1 × 10^6^) were plated in a 6-well plate. Subsequently, cells were serum starved for 24 hours and the media were collected. Cells (2.5 × 10^5^) were serum starved for 24 hours and pre-treated for 30 minutes with the following drugs prior to stimulation with 100 nM KP-10 for 24 hours: 20 μM active ERK1/2 inhibitor U0126 (Millipore), 20 μM inactive analog U0124 (Millipore) [79], or vehicle dimethyl sulfoxide (DMSO). Media were then collected, and samples were centrifuged at 350 rcf for 5 minutes to remove cellular debris. Samples were combined 1:1 with sample buffer (0.5 M Tris-HCl pH 6.8, 10% SDS, glycerol, 1% bromophenol blue) and 40 μL of sample was loaded onto a 10% SDS-PAGE separating gel copolymerized with 0.1% gelatin. The gels were then washed in 2.5% Triton X-100 to remove the SDS and renature the MMP proteins. The gels were incubated for 48 hours at 37° C with gentle agitation in developing buffer (1 M Tris, 5 M NaCl, 1 M CaCl2, 30% Brij-35, pH 7.4) to allow enzymatic digestion of the gelatin. Following incubation, gels were stained with Coomassie blue, de-stained, and imaged using the VersaDoc imaging system.

### MTT assays

MTT cell viability assays were conducted as previously described [37]. MDA-MB-231 and Hs578T cells expressing fibulin-3 shRNA or scrambled control were plated in triplicates (5 × 10^4^) on a 96-well plate. MTT labeling reagent was added to each well (final concentration 0.5 mg/ml) and cells were incubated for 4 hours at 37° C and 5% CO_2_, solubilized in DMSO, and absorbance was measured using a Victor 3V Multi-Detection Microplate Reader (PerkinElmer).

### Cell growth assays

To determine if shRNA expression had any impact on cell growth rate, MDA-MB-231 and Hs578T cells (4 × 10^5^) expressing either fibulin-3 shRNA or scrambled control were plated in 60 mm culture plates, as described [37]. Cells were trypsinized and counted using a haemocytometer at 24-hour intervals.

## SUPPLEMENTARY MATERIALS FIGURES AND VIDEO





## Abbreviations

EFEMP1: epidermal growth factor (EGF)-containing fibulin-like extracellular matrix protein 1
ECM: extracellular matrix
EMT: epithelial-to-mesenchymal-transition
ERα: estrogen receptor α
KISS1R: kisspeptin 1 receptor
KP-10: kisspeptin peptide-10
KISS1: kisspeptin peptide-145
MT1-MMP: membrane type 1 matrix metalloproteinase
MMP-9: matrix metalloproteinase 9
MAPK: mitogen-activated protein kinase
TNBC: triple negative breast cancer
