# Supplementary material for: The matrix protein Fibulin-3 promotes KISS1R induced triple negative breast cancer cell invasion
Source: Oncotarget. 2018 Jul 10;9(53):30034–52. doi: 10.18632/oncotarget.25682 (PMC6059025; doi:10.18632/oncotarget.25682)
Supplement: Supplementary file 1 [file oncotarget-09-30034-s001.pdf]

The matrix protein Fibulin-3 promotes KISS1R induced triple negative breast cancer cell invasion

SUPPLEMENTARY MATERIALS

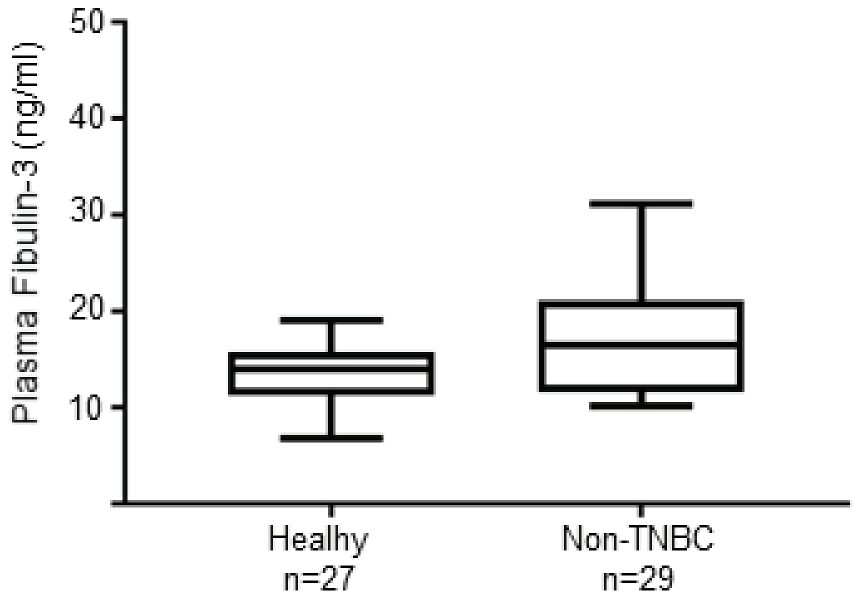

Supplementary Figure 1: Fibulin-3 expression levels in non-TNBC (ER/PR-positive, HER2-negative) patients. Plasma fibulin-3 levels (ng/mL) measured by ELISA in blood samples taken from healthy subjects (n = 27), or non-TNBC breast cancer patients (n = 29). Statistical analysis done using Wilcoxon two-sample test. Error bars: SD.

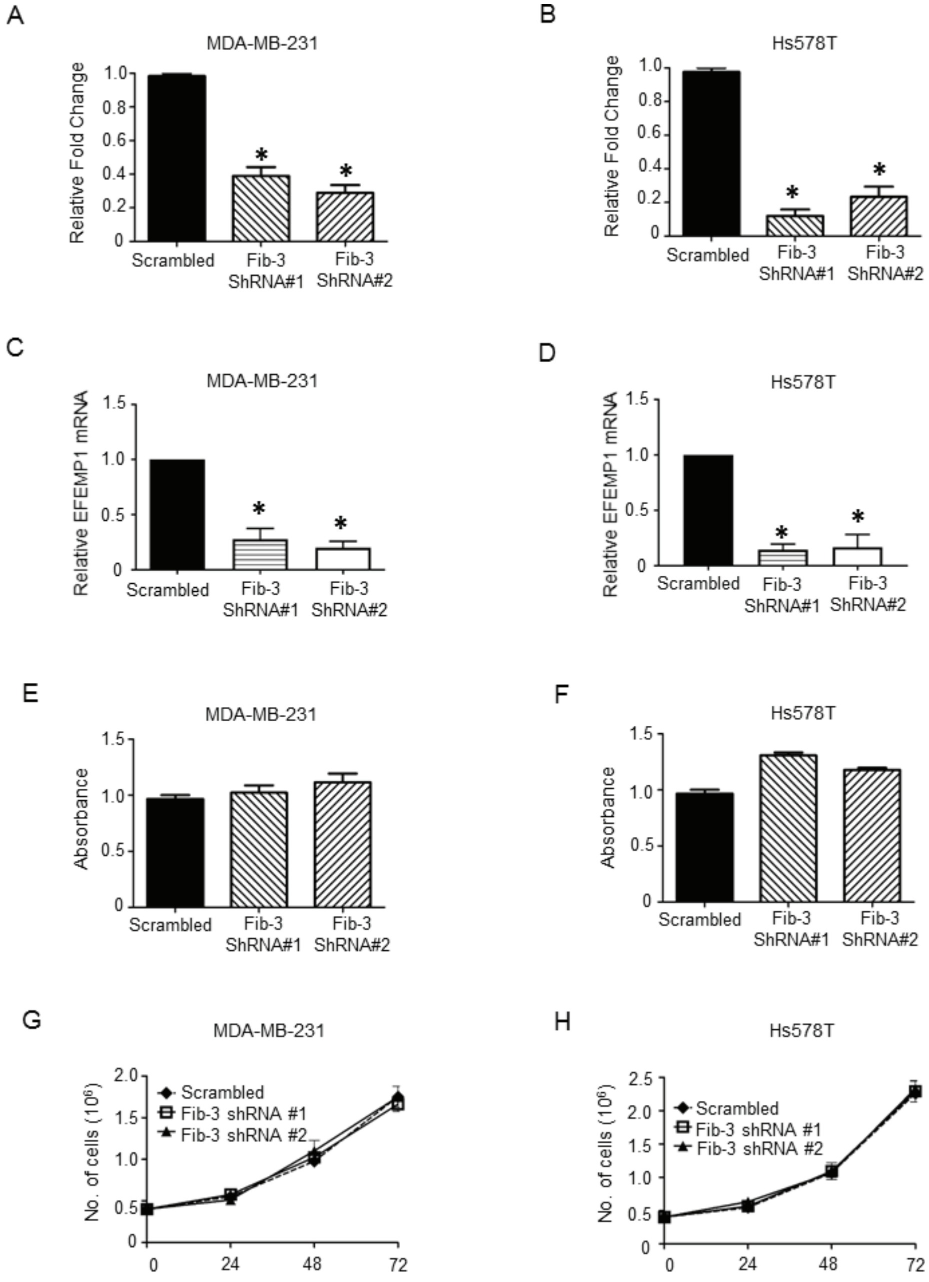

Supplementary Figure 2: Fibulin-3 knockdown in TNBC cells. Densitometric analysis of western blots (representative shown in Figure 2A) of the expression of fibulin-3 in (**A**) MDA-MB-231 and (**B**) Hs578T cells stably expressing fibulin-3 shRNA or scrambled control (n = 3). One-way ANOVA followed by Dunnett's multiple comparison test: $^{*}P < 0.05$ compared to scrambled. Bars represent protein expression ± SEM. (**C**) Relative mRNA expression of fibulin-3 gene, *EFEMP1* by RT-qPCR in (C) MDA-MB-231 and (**D**) Hs578T cells stably expressing fibulin-3 shRNA or scrambled control (n = 3). Columns represent mean relative mRNA expression, normalized to GAPDH ± SEM; student's unpaired T-test: $P < 0.05$. Cell viability assays of (**E**) MDA-MB-231 and (**F**) Hs578T cells expressing fibulin-3 shRNA or scrambled control cultured in 96-well plates as determined by MTT assay (n = 3). Bars represent absorbance measured at 550 nm minus background reading at 700 nm ± SEM. MDA-MB- 231 (**G**) or Hs578T (**H**) cells (4×10^5) expressing fibulin-3 shRNA or scrambled control were cultured for 72 hours and cells counted at 24-hour intervals (n = 3); downregulation of endogenous fibulin-3 does not affect cell growth.

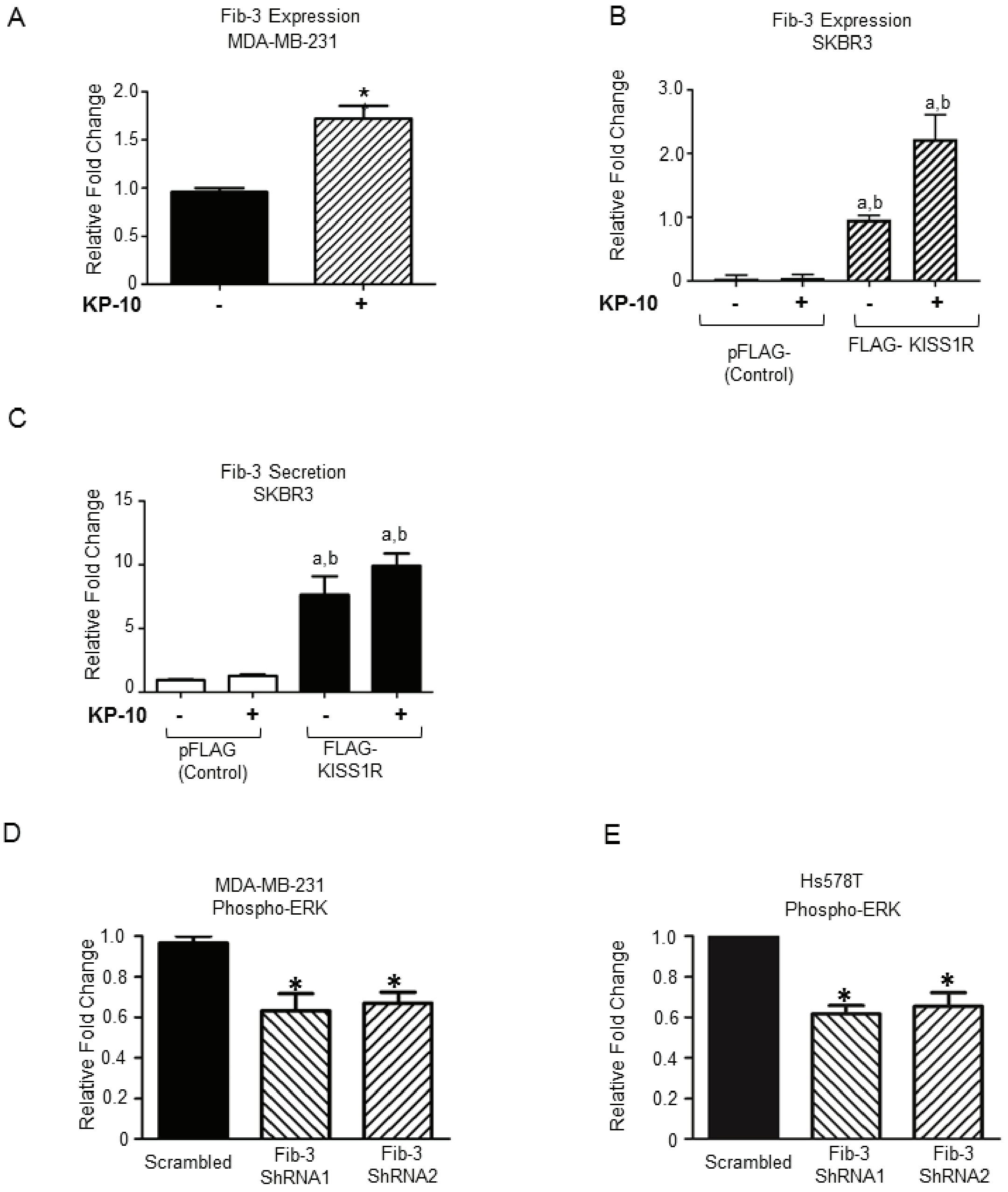

Supplementary Figure 3: Densitometric analysis of western blots. (**A**) Densitometric analysis of western blots showing the expression of fibulin-3 in MDA-MB-231 cells treated with KP-10 (100 nM, 72 h; see Figure 5D for representative blot; $n = 3$). Bars represent protein expression ± SEM. *Student's *t*-test: $P < 0.05$ compared to unstimulated cells. (**B**) Densitometric analysis of western blots showing fibulin-3 expression in SKBR3 cells stably expressing FLAG-KISS1R (see Figure 5D for representative blot; $n = 3$). One-way ANOVA followed by Dunnett's multiple comparison test: a, $P < 0.05$ compared to pFLAG vehicle; b, $P < 0.05$ compared to pFLAG KP-10. Bars represent protein expression ± SEM. (**C**) Densitometric analysis of western blots showing fibulin-3 expression in conditioned media from SKBR3 cells stably expressing FLAG-KISS1R or pFLAG vector control following KP-10 (100 nM, 72 h treatment; $n = 3$; see Figure 5E for representative blot). One-way ANOVA followed by Dunnett's multiple comparison test: a, $P < 0.05$ compared to pFLAG vehicle; b, $P < 0.05$ compared to pFLAG KP-10. Bars represent protein expression ± SEM. Densitometric analysis of western blots in Figure 7A, 7B showing pERK expression in (**D**) MDA-MB-231 cells or (**E**) Hs578T cells expressing fibulin-3 shRNA or scrambled control ($n = 3$). One-way ANOVA followed by Dunnett's multiple comparison test: $^{*}P < 0.05$ compared to scrambled. Bars represent protein expression ± SEM.

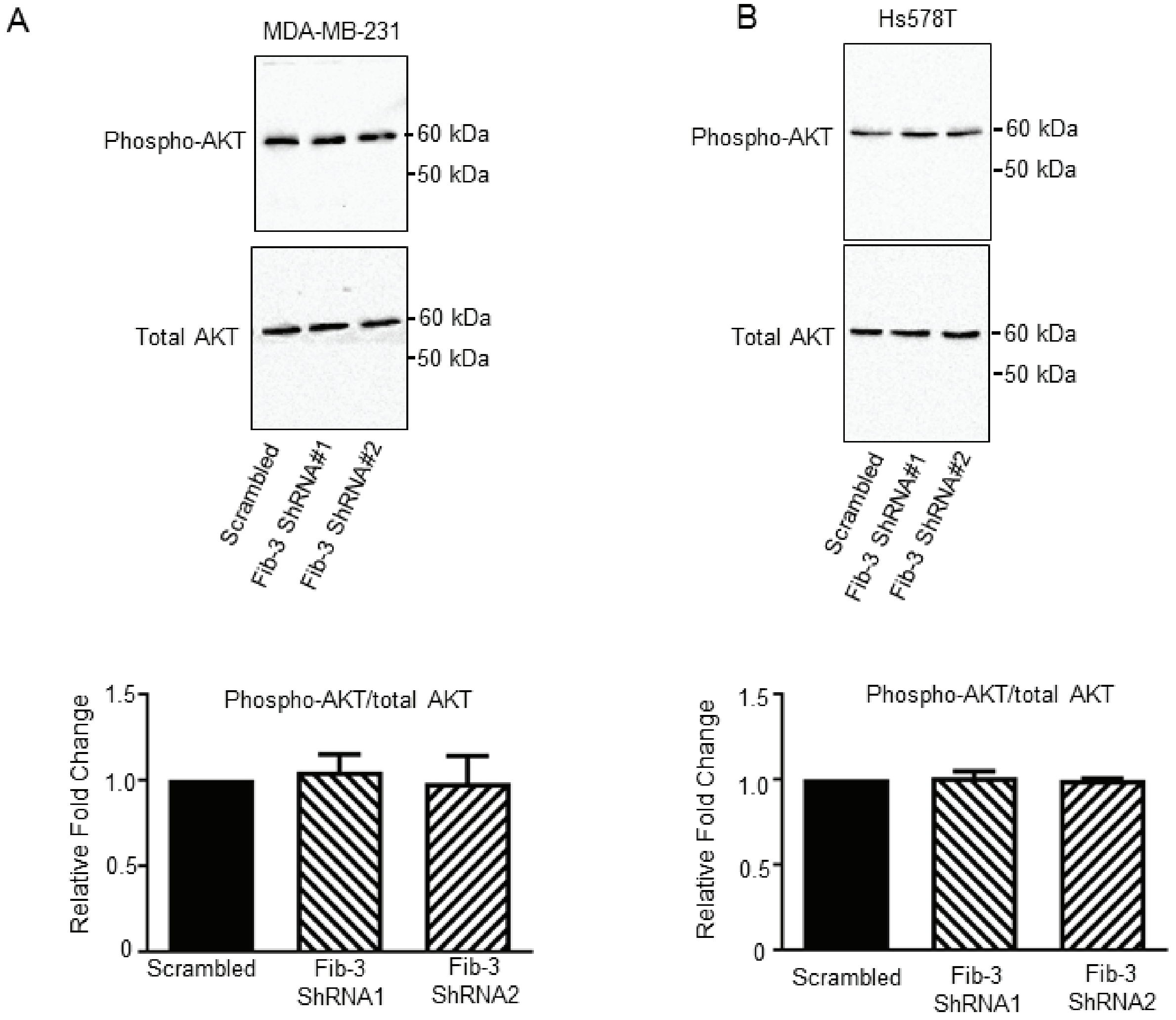

Supplementary Figure 4: Western blots of AKT expression in TNBC cells. Representative western blot of pAKT and total AKT in (**A**) MDA-MB-231 cells or (**B**) Hs578T cells, expressing fibulin-3 shRNA or scrambled control; total AKT, loading control ($n = 4$). Bars ± SEM represent protein expression in the densitometric analysis of blots.

Supplementary Movie 1: Time-lapse microscopy of MDA-MB-231 cells stably expressing scrambled control or fibulin-3 shRNA (top and bottom panels, respectively); images acquired every 15 minutes for 18 h using Olympus IX-81 microscope. See Supplementary_Movie_1
